# Hybrid surgery for coexistence of cerebral arteriovenous malformation and primitive trigeminal artery: A case report and literature review

**DOI:** 10.3389/fsurg.2022.888558

**Published:** 2022-07-26

**Authors:** Lesheng Wang, Jieli Li, Zhengwei Li, Songshan Chai, Jincao Chen, Nanxiang Xiong, Bangkun Yang

**Affiliations:** ^1^ Department of Neurosurgery, Zhongnan Hospital of Wuhan University, Wuhan, China; ^2^ Brain Research Center, Zhongnan Hospital of Wuhan University, Wuhan, China

**Keywords:** primitive trigeminal artery, cerebral arteriovenous malformation, hybrid surgery, case report, literature review

## Abstract

The primitive trigeminal artery (PTA), an abnormal carotid-basilar anastomosis, forms the vascular anomaly connection between the internal carotid artery and vertebrobasilar system. Rarely, PTA can be complicated by several other cerebrovascular disease, including arteriovenous malformations (AVMs), intracranial aneurysms, moyamoya disease, and carotid-cavernous malformations. Herein, we reported a rare case of PTA combined with an AVM in a male patient. The patient was a 28-year-old male with epileptic seizures at the onset of symptoms. Magnetic resonance imaging showed abnormal signal foci and localized softening foci formation with gliosis in the right parietal temporal lobe. Furthermore, using a digital subtraction angiogram (DSA), it was found that an abnormal carotid-basilar anastomosis had developed through a PTA originating from the cavernous portion of the right internal carotid artery (ICA) and a large AVM on the surface of the right carotid artery. The lesion of AVM tightly developed and draining into superior sagittal sinus. A hybrid operating room was used for the surgery. The main feeding arteries of the AVM originating from three major arteries, including the right middle cerebral artery, the right anterior cerebral artery, and the right posterior cerebral artery, were clipped and subsequently, then the AVM was thoroughly removed. The intraoperative DSA showed that the AVM had been resected completely. Postoperative pathological examination of the resected specimen indicated the presence of an AVM. The patient recovered well after surgery and has been symptom-free for more than 3 months. In summary, the pathogenesis of the coexistence of PTA and AVM remains unknown. As highlighted in this case report, hybrid surgery can be used to remove AVMs and can improve the patients' prognosis. To our best knowledge, this is the first case in the literature of successful AVM treatment using hybrid surgery.

## Introduction

The primitive trigeminal artery (PTA), also known as the persistent trigeminal artery is a relatively rare vascular anomaly characterized by the embryonic arteries that connect the internal carotid artery (ICA) to the vertebrobasilar system (1). To date, PTA has an incidence of 3–22 cases per 10,000 people (2) and an estimated incidence of 0.1%–1.0% on cerebral angiograms (3). Although the incidence of PTA is not high at present, it is commonly combined with other cerebrovascular diseases, including cerebral aneurysms (4–6), cavernous sinus fistula (7–9), trigeminal neuralgia (10, 11), and cerebral arteriovenous malformation (AVM) (12–17).

PTA is associated with a higher incidence of AVMs (approximately 4.5%) (14). Generally, patients who undergo PTA alone do not require surgery or other treatments. Other comorbidities including cerebral aneurysms and AVMs, require different management strategies and treatments. The prognosis of most patients is considered to be good. However, there have been relatively few reports of coexistence of AVMs and PTA. Herein, we present a rare case of temporal AVM with PTA in a patient who underwent hybrid surgery.

## Case report

**Figure 1:**
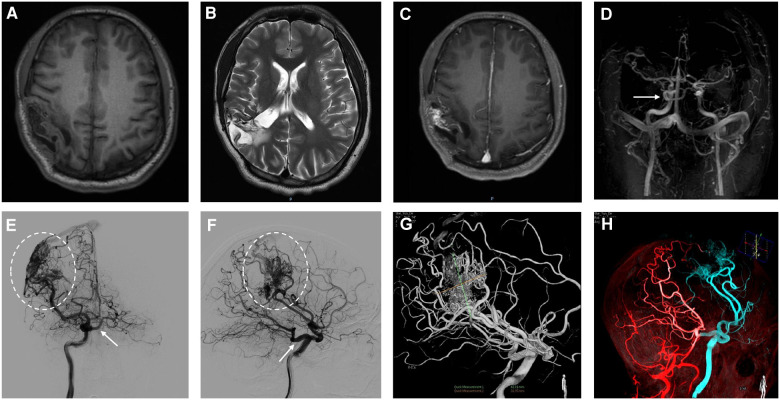
In the right parietotemporal lobe, an irregularly circular space-occupying lesion was found with slightly long T1 shadows (**A**). T2-weighted image shows a parietotemporal lesion with mixed signal characteristics (**B**). Enhanced MRI shows heterogeneous enhancement in the right parietotemporal lobe (**C**). The primitive trigeminal artery arose from the cavernous sinus segment of the right ICA (**D**). Initial right internal carotid artery injections, anteroposterior (**E**) and lateral views (**F**), show shunted flow to the cavernous sinus (arrow). Anteroposterior and lateral projection angiogram shows a right parietotemporal AVM supplied by the right middle cerebral artery drainage into the superior sagittal sinus (**E,F**, dashed oval). 3-dimensional (3D) DSA arterial phase indicates in the lateral projection showing AVM lesion with 42.8 mm in size and PTA obtained after right internal carotid artery injection (**G**). Fused image of 3D DSA demonstrates the flow of the right ICA (blue), VA (red) (**H**).

A 28-year-old male presented with sudden cerebral hemorrhage 5 years ago and underwent decompressive hemicraniectomy with hematoma evacuation. The patient recovered into a good condition after surgery. The patient experienced grand mal epileptic seizures at 5 years after surgery. The patient was treated with antiseizure medication and showed poor response. Following the onset of symptoms, the patient was admitted to our hospital. The right limbs moved freely, while the left limbs were reflexive (muscle strength grade IV). The positive Babinski sign was presented in the left lower limb. Magnetic resonance imaging showed abnormal signal foci and localized softening foci formation with gliosis in the right parietotemporal lobe (Figures 1A–C). Magnetic resonance angiography (MRA) indicated carotid-basilar anastomosis and the presence of vascular malformations in the right parietal temporal lobe (Figure 1D). Further evaluation using DSA revealed an abnormal anastomosis between the carotid and basilar arteries through a PTA originating from the cavernous portion of the right internal carotid artery (ICA) (Figures 1E–H). Vascular malformation is tight, and a large number of fine branching arteries from the frontotemporal branch of the right middle cerebral artery, the right anterior cerebral artery, the right posterior cerebral artery, and the right external carotid artery served as its blood supply (Figures 1E–H). The maximum size of the AVM was approximately 42.8 mm.

**Figure 2:**
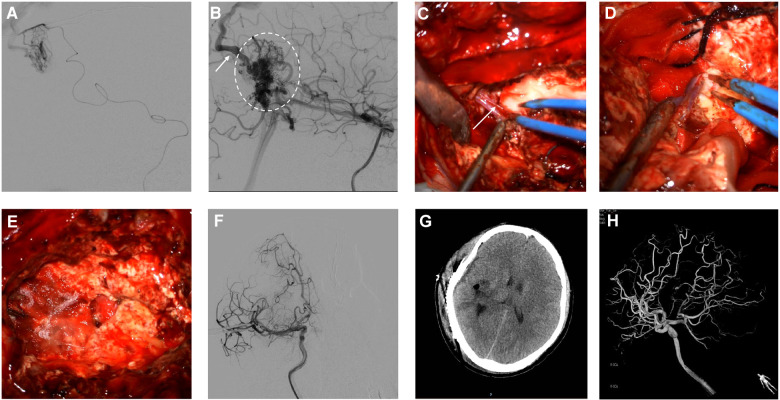
Intraoperative angiography demonstrates subsequent injection of Onyx18 with obliteration of the AVM (**A**). Lateral view of right ICA angiogram demonstrates the presence of residual arteriovenous malformation (dashed oval) with an early draining vein (arrow) (**B**). The feeding artery arteries and main draining veins were isolated and resected (**C,D**, arrows). Intra-operative image shows lesion excised during surgery (**E**). Postoperative anteroposterior projection angiograms following resection demonstrate the obliteration of the AVM (**F**). Postoperative Contrast CT shows no obvious significant ischemic or hemorrhage changes (**G**). Follow-up 3D DSA shows the complete disappearance of AVM (**H**).

Headaches and dizziness restricted the patient's daily activities; therefore, he underwent hybrid surgery to completely resect the AVM. AVM resection was scheduled to be performed in a hybrid operating room. AVM resection and preoperative embolization were performed in a single stage. Emboli were performed *via* endovascular and intravascular embolization using a biplane flat-panel angiographic suite (UNIQ FD2020 Hybrid-OR, Philips, Eindhoven, the Netherlands) and 3D reconstruction under general anesthesia. After the incision of the skin, a 6-F guiding catheter (Medtronic, Irvine, CA, USA) was placed in the right femoral artery by a percutaneous puncture. The presurgical embolization procedure involved identifying the main supplying artery of the AVM, guiding superselective cannulation into the feeding artery with a microcatheter system, embolizing the vessels with an ethylene-vinyl copolymer (Onyx 18, Medtronic, Inc., Minneapolis, Minnesota, USA), and confirming continued injection until the feeding artery stasis (Figure 2A). Intraoperative DSA showed a residual AVM with an early draining vein (Figure 2B). Subsequently, a right-expanded subtemporal approach was adopted during surgery. The AVM is located on the surface of the right parietal temporal lobe. The AVM's major feeding artery was clipped, which arose from the right middle cerebral artery, the right anterior cerebral artery, and the right posterior cerebral artery (Figure 2C). The dura was cut under a microscope and observed to be tightly adherent to the brain surface tissue (Figure 2D). The lesion in the AVM was removed afterwards, resulting in obsolete hemorrhage in the AVM. There was an AVM with a tight venous end draining into the superior sagittal sinus. No focal regions were observed in the right hemisphere (Figure 2E). The final pathological examination after removal showed an irregular vascular shape consisting of various types of expanded and transparent veins and abnormal muscularized arteries. DSA was performed immediately after the AVM resection, which showed that the AVM was completely removed (Figure 2F). The patient recovered well postoperatively without complications. A 3e-day postoperative computed tomography scan showed good recovery of brain tissue (Figure 2G). The patient was in good condition without any episodes of seizures, had grade IV muscle strength in the left limb, and could take care of himself. On postoperative DSA rightward head rotation, the AVM lesion completely disappeared (Figure 2H).

## Discussion

The PTA ranging from 0.1% to 0.6%, is an embryological anastomosis between the cavernous sinus segment of the ICA and the basilar artery and is the most common anomalous traffic between the ICA system and the vertebrobasilar system (18). Other anomalous anastomoses include the persistent subungual, auditory, and intersegmental arteries. The etiology of PTA may be due to the non-degeneration of the anastomosing branch between the embryonic aorta and the neural artery during the embryonic period; normally this vessel degenerates after the development of the posterior communicating artery. Some authors have suggested that the failure of trigeminal artery degeneration is due to occlusion of the proximal portion of the fetal internal carotid artery, resulting in the inevitable persistence of the trigeminal artery to maintain an adequate blood supply to the forebrain by retrograde transport of blood from the basilar artery to the carotid artery (19).

PTA was first proposed by Saltzman in 1958 and is divided into two main types (20). In Saltzman type 1, PTA supplies the vessels distal to the anastomosis, the posterior communicating arteries bilaterally, and the basilar arteries below the junction of the PTA and the basilar arteries are hypoplastic or absent. In Saltzman type II, PTA supplies the superior cerebellar arteries bilaterally, and the posterior cerebral arteries bilaterally are supplied by the ipsilateral posterior communicating arteries. Previous studies have indicated that the relative incidence of Saltzman types 1 and 2 is approximately equal (21). However, the typology proposed by Saltzman is rather general and does not elaborate on the anatomical features of PTA. Therefore, in 2011, Weon et al. proposed a new typology based on the Saltzman typology, namely the Weon typology (22). The definitions of types I and II remain consistent with those described by Saltzman et al. In the Weon type III, PTA supplies the contralateral posterior cerebral artery, and the ipsilateral posterior cerebral artery is supplied by the posterior communicating artery. In the Weon type IV, PTA supplies the ipsilateral posterior cerebral artery, and the posterior communicating artery supplies the contralateral posterior cerebral artery. In the Weon type V, all other variants of PTA, including those ending in the superior anterior inferior, and posterior inferior cerebellar arteries. Hence, according to the classification suggested by Weon et al., the case we reported belongs to Weon type V.

Associations between PTA and cerebrovascular diseases, especially with vertebrobasilar embolic ischemia, and with vascular nerve compression syndrome, have been reviewed (23–25). These could not be confirmed in large-scale studies and, therefore, represent more likely coincidental findings rather than true associations in the absence of other arterial vascular anomalies or syndromes (18). However, the pathogenesis underlying the coexistence of PPTA and MMD remains unknown.

To our knowledge, only eight reported cases of AVM have occurred in association with PTA (Table 1) (12–14, 16, 26–29). In six out of the eight cases, the patients received different treatments, including microsurgery, conservative treatment, radiosurgery, embolization, and radiosurgery combined with embolization. In the remaining two cases, although the requested original texts could not be found, the good outcomes of the patients were described in their abstracts. Considering these results, the intervention would be the first option for AVMs associated with a PTA when patients have indications of treatment. In our case, hybrid surgery was performed to remove the lesion, and the patient recovered well after the operation.

**Table 1:** Summary of AVM associated with PTA.

No.	Author, year	Age/Gender	Clinical Presentation	Location of PTA	Location of AVM	Treatment	Outcome
1	Jayaraman, 1977	27/Female	Subarachnoid hemorrhage	Left side	Left superior temporal lobe	Uncertain	Good
2	Uchino, 1989	16/Female	Sudden onset of severe headache and vomiting	Left side	Left Frontal lobe	Radiosurgery	Good
3	Matsko, 1991	22/Female	Unknown	Unknown	Unknown	Embolization	Good
4	Takumi, 1994	48/Female	Sudden loss of consciousness	Left side	Right parietal lobe	Microsurgery	Good
5	Nakai, 2000	58/Male	Sudden onset of headache and vomiting	Left side	Cerebellum	Conservative management	Good
6	Ohtakara, 2000	21/Female	Wallenberg's syndrome and Foville's syndrome	Left side	Brain stem and left cerebellum	Embolization and radiosurgery	Good
7	Igor, 2011	31/Female	Subarachnoid hemorrhage and cerebellar hematoma	Left side	Cerebellum	Microsurgery	Good
8	Kenichi, 2013	53/Male	Trigeminal neuralgia	Left side	Cerebellum	Embolization	Good
9	Present case	28/male	Hemmorrahge	Right side	Right temporo-parietal lobe	Hybrid surgery	Good

Recently, multimodality treatment, especially hybrid surgery, has received increasing attention as an effective treatment for intracranial AVMs (30, 31). Preoperative partial embolization of the malformation can assist surgical positioning, reduce blood flow to the malformation, and reduce the risk of intraoperative bleeding risk and surgical difficulty (32). Intraoperative angiography can detect the residual malformation immediately after one-stop resection, greatly reducing the residual rate of postoperative malformation and the risk of postoperative rebleeding (33–36). Despite the lack of a large number of randomized controlled trials have reported that the application of a hybrid operating room provides more satisfactory effectiveness than traditional surgery.

## Conclusion

In summary, coexistence of AVM and PTA was successfully treated with hybrid surgery. A one-stop hybrid operation combining embolization and microsurgical resection could be performed as a safe and effective intervention strategy for AVMs. More follow-up data is required to determine the long-term effects of surgery.

## Data Availability

The raw data supporting the conclusions of this article will be made available by the authors, without undue reservation.
